# Computer-Aided Chemotaxonomy and Bioprospecting Study of Diterpenes of the Lamiaceae Family

**DOI:** 10.3390/molecules24213908

**Published:** 2019-10-30

**Authors:** Andreza Barbosa Silva Cavalcanti, Renata Priscila Costa Barros, Vicente Carlos de Oliveira Costa, Marcelo Sobral da Silva, Josean Fechine Tavares, Luciana Scotti, Marcus Tullius Scotti

**Affiliations:** Post-Graduate Program in Natural Synthetic Bioactive Products, Federal University of Paraiba, João Pessoa 58051-900, Paraíba, Brazil; andreza.jp.pb@gmail.com (A.B.S.C.); vicente@ltf.ufpb.br (V.C.d.O.C.); marcelosobral.ufpb@gmail.com (M.S.d.S.); josean@ltf.ufpb.br (J.F.T.); luciana.scotti@gmail.com (L.S.)

**Keywords:** Lamiaceae, database, diterpenes, chemotaxonomic, SOMs

## Abstract

Lamiaceae is one of the largest families of angiosperms and is classified into 12 subfamilies that are composed of 295 genera and 7775 species. It presents a variety of secondary metabolites such as diterpenes that are commonly found in their species, and some of them are known to be chemotaxonomic markers. The aim of this work was to construct a database of diterpenes and to use it to perform a chemotaxonomic analysis among the subfamilies of Lamiaceae, using molecular descriptors and self-organizing maps (SOMs). The 4115 different diterpenes corresponding to 6386 botanical occurrences, which are distributed in eight subfamilies, 66 genera, 639 different species and 4880 geographical locations, were added to SistematX. Molecular descriptors of diterpenes and their respective botanical occurrences were used to generate the SOMs. In all obtained maps, a match rate higher than 80% was observed, demonstrating a separation of the Lamiaceae subfamilies, corroborating with the morphological and molecular data proposed by Li et al. Therefore, through this chemotaxonomic study, we can predict the localization of a diterpene in a subfamily and assist in the search for secondary metabolites with specific structural characteristics, such as compounds with potential biological activity.

## 1. Introduction

Historically, natural products have been used as sources to treat, cure and prevent diseases [1]. The greatest contribution of these natural products occurs through plants, which can be classified according to their chemical constitution, and this classification is defined as chemotaxonomy. A wide variety of studies include the chemotaxonomic classification of secondary metabolites; among the most investigated compounds are phenolics, alkaloids, terpenoids and nonprotein amino acids [2].

Lamiaceae is one of the largest families of Angiosperms, the largest family of Lamiales, an order comprising 26 families and more than 20,000 species [3]. This family is classified into 12 subfamilies, which are composed of 295 genera and 7775 species [4]. Their species are usually represented by herbs and shrubs that are distributed throughout the world in tropical and temperate regions [3,5].

This family accumulates substances with very diverse structures and many of them are reported as chemotaxonomic markers at all levels: subfamily, genus and species [6]. The main secondary metabolites isolated from Lamiaceae species are monoterpenes [7], sesquiterpenes [8], diterpenes [9], triterpenes [10], pyrones [11], iridoids [12], phenolic compounds [13] and flavonoids [14]. Among these, diterpenes are more prominent as chemotaxonomic markers because they are easily found in most species of this family [6,15,16,17,18,19,20].

Several botanical studies have demonstrated the classification of Lamiaceae in the level of subfamilies. In the work of Harley et al. [21], it was observed that 236 genera are distributed in seven subfamilies: Ajugoideae, Lamioideae, Nepetoideae, Prostantheroideae, Scutellarioideae, Symphorematoideae and Viticoideae, although 10 genera were left unassigned at the subfamily level.

**Figure 1:**
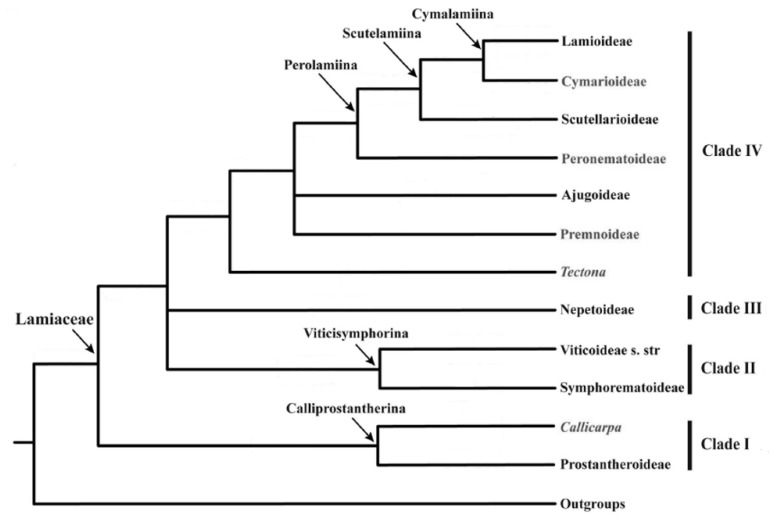
Phylogenetic diagram of Lamiaceae subfamilies (adapted from Li et al. [3]).

Recently, Li et al. [3] presented a review containing several findings that strengthen arguments for a new classification of the family Lamiaceae, reporting for each subfamily its phylogenetics and morphology. They observed through DNA analysis the presence of three new subfamilies, making up a total of 10 subfamilies (Figure 1). Of the ten genera that were unclassified in the study by Harley et al. [21], the only two that were not allocated to a subfamily were *Callicarpa* and *Tectona*. However, these two genera are inserted between the subfamilies in positions that corroborate with the phylogeny (Figure 1).

In the study by Li et al. [22], the presence of two new subfamilies, Callicarpoideae and Tectonoideae, was determined, which in the previous study had not been classified. Therefore, the current Lamiaceae classification is composed of 12 subfamilies arranged in four clades, thus facilitating the organization of genera and species [3,22].

The biological and physicochemical properties of the molecules can be predicted through molecular descriptors, which are the result of the conversion of the symbolic representation of a chemical structure into a useful number [23], and there are several software packages to generate molecular descriptors, such as Dragon 7.0 [24]. The descriptor can be used to obtain chemical patterns that, to be visualized, need the creation of computational models that can be obtained using several algorithms such as the use of artificial neural networks (ANNs).

ANNs are defined as a mathematical model inspired by the neural structure of intelligent organisms, in which several nodes, called neurons, are interconnected in a network-like structure [25,26]. In the process of identifying and classifying patterns, the commonly used ANNs’ unsupervised architecture is the self-organizing map (SOM). This is an unsupervised method capable of providing multivariate data maps in a two-dimensional (2-D) grid. It results in the clustering of similar patterns next to each other and has been used successfully in different studies that use database chemistry, including chemotaxonomic studies [25,26,27,28,29,30,31].

In the search for secondary metabolite banks already isolated from the Lamiaceae family, we can use databases that provide information about the compounds, such as biological, biogeographical and taxonomic data [32]. Some of these tools are commercially available or freely available, such as the Bioassay Nucleus, Biosynthesis and Ecophysiology of Natural Products (NuBBE) [33], Dictionary of Natural Products (DNP) [34], NAPRALERT [35] and Marinlit for natural marine products [36].

SistematX has a different relationship to the other databases available on the web, in that it is possible to use a browser to directly add and manage the data useful to the academic community about the secondary metabolites, such as research by chemical structure, SMILES code, compound names as well as information-specific species for taxonomic classification (from family to species) and the geographic location of the species from which the compounds were isolated [32].

Thus, the aim of this work is to construct a database of diterpenes from the Lamiaceae family and extract information for chemotaxonomic analysis among the subfamilies, using the molecular descriptors and SOMs, and comparing the results with the phylogenetic classification proposed by Li et al. [3]. This will test if it is possible to predict the botanical occurrence in its corresponding subfamily.

## 2. Results and Discussion

### 2.1. Database

The database is composed of diterpenes isolated from species of the family Lamiaceae; it comprises 4115 different chemical structures and corresponds to 6386 botanical occurrences and 4880 geographical locations. The number of occurrences for a superior taxon is defined counting how many times a compound appears in determined species belonging to that taxon. All data are available in the SistematX tool (https://sistematx.ufpb.br). As shown in Table 1, the 4115 diterpene molecules are distributed in eight subfamilies, 66 genera and 639 different species of the Lamiaceae family. The subfamily Nepetoideae presents the greatest number of genera, species and botanical occurrences. Of the total number of botanical occurrences, only seven botanical occurrences were unclassified at subfamily level, therefore totaling 6379.

### 2.2. Self-Organizing Maps and Molecular Descriptors Applied in the Chemotaxonomy of Lamiaceae Subfamilies

From the botanical occurrences of the diterpenes obtained from the Lamiaceae family, 108 molecular descriptors were generated for each molecular structure using Dragon 7.0 software [24]. The botanical occurrences were classified into four subfamilies and the values of the descriptors were used as input data for the SOM Toolbox 2.0 software [37]. The subfamilies selected for analysis were those that presented the highest number of botanical occurrences making possible the pattern recognition of the distribution of diterpenes in Lamiaceae (Table 1). Then, the self-organizing matrix for each molecule was calculated, dividing the samples into groups according to the similarity and after comparing the SOM with the classification proposed by Li et al. [3].

In the maps depicted, the chemical occurrences of certain subfamilies occupy regions that are labeled by the following colors:

1. Clade III (Nepetoideae), red;
2. Clade IV (Ajugoideae, Lamioideae and Scutellarioideae), lilac;
3. Ajugoideae, blue;
4. Lamioideae, green;
5. Scutellarioideae, dark blue.

The SOM that was obtained using the occurrences of the diterpenes of clade III (Nep) and clade IV (Aju, Lam and Scu) subfamilies showed a total hit rate of 86.3%, with 6025 occurrences and 5200 hits (Table 2). The SOM generated using fingerprint to analyze the correspondence of botanical occurrences of clade III and clade IV subfamilies resulted in a total hit rate of 89.5%. These data corroborate a good separation of the subfamilies because even though different descriptors were used, the results were similar (Table 2).

**Figure 2:**
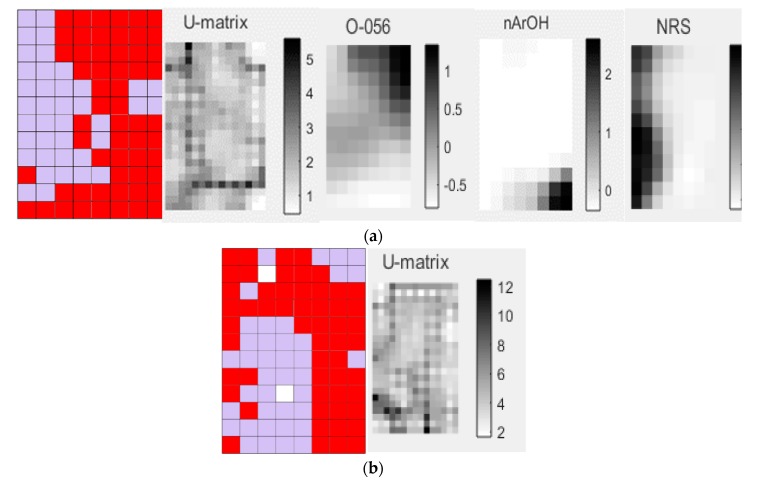
Self-organizing map obtained by classification of the subfamilies of clade III (red) and clade IV (lilac) and generated descriptors: (**a**) SOM → molecular descriptors; U-matrix; O-056, nArOH and NRS. (**b**) SOM → fingerprint and U-matrix.

The SOM (Figure 2) shows a clear separation between the botanical occurrences of clade III (red) and clade IV (lilac), reaffirming the phylogenetic analysis performed by Li et al. (Figure 1) [3]. Analyzing the SOM, there is a chemical pattern that shows a region in which the subfamily Nep (red) occupies many neurons distributed by the map, being the one with the highest number of occurrences (3644) and the best rate of success 89.2% (Table 2). The predictive performance of the SOM for the five training and test sets that were generated from the original set can be visualized in Table 3. The applicability domain (AD) was reliable for more than 99% of the predictions of the test set. The average match rate for the five test sets (85.4%) is very close to that of the training (86.4%). The clade III (Nep subfamily) shows the highest match rate values for training sets (88.6%) and tests (88.3%), while clade IV (subfamilies Aju, Lam and Scu) showed 82.1% and 81% for training and test sets, respectively.

Chemotaxonomy analysis was also performed using other machine learning algorithms: support vector machine (SVM), which is a supervised machine learning algorithm, and k- nearest neighbors (k-NN), which is an instance-based algorithm. Results are shown in Table 4 for the analysis performed on the SOM by clade. It can be observed that, as in the SOM, the models generated with SVM and k-NN obtained very similar results and with high hit rates.

The applicability domain (AD) was reliable for over 99% of the test set predictions for all algorithms used: SOM with molecular descriptor, SOM with fingerprint, SVM and k-NN.

The most significant descriptors for the clustering the diterpenes of the Ajugoideae, Lamioideae, Scutellarioideae (clade IV) and Nepetoideae (clade III) subfamilies are also shown in Figure 2. The U-matrix shows the distances between the neighboring map unit, where high values indicate a border of a cluster and uniform areas of low values indicate the clusters themselves (Figure 2a). The subfamily of clade III shows a high value for the following descriptors, which are shown in black in Figure 2a: atoms-centered descriptor O-056 that encodes alcohol and functional group count nArOH that encodes the number of aromatic hydroxyls. The diterpenes of the clade IV subfamilies present high values for the ring descriptor NRS that encodes the number of ring systems (Figure 2a).

**Figure 3:**
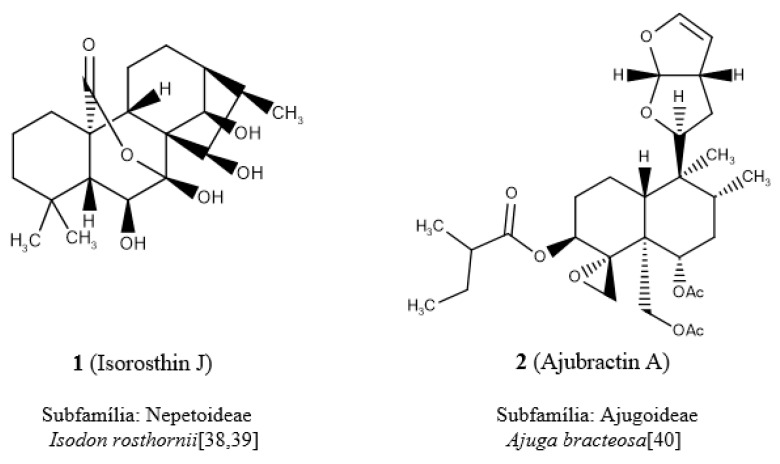

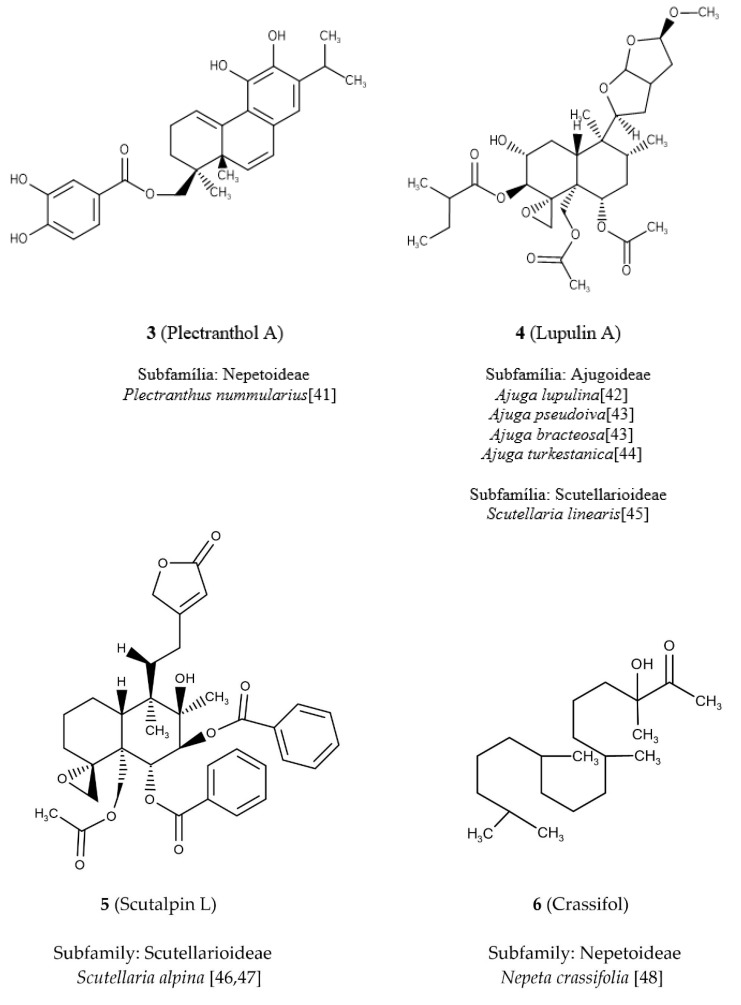
Chemical structures of the diterpenes located in the SOM of clade III (Nep) and clade IV (Aju, Lam, Scu) and their respective botanical occurrences.

In analyzing the individual descriptors, it was verified in the descriptor of atom-centered fragments, O-056 (alcohol), that its highest value was attributed to diterpene **1** (Figure 3) due to the presence of four alcohols. This diterpene is popularly known as isorosthin J [38,39] and belongs to the subfamily Nepetoideae (clade III). The diterpene **2** (Figure 3), known as ajubractin A [40], belongs to the subfamily Ajugoideae (clade IV) and presents the null value for the descriptor O-056. It was observed that diterpene **3** (Figure 3), known as plectranthol A [41], has the highest value of the nArOH descriptor, with the presence of four aromatic hydroxyls, whereas the lowest value, null, for this descriptor was attributed to diterpene **4**, lupulin A [42,43,44,45] (Figure 3).

It was reported in the literature that plectranthol A (**3**) shows antioxidant activity [41] and, according to this chemotaxonomic study, it is observed that it can be found in a species belonging to the subfamily Nepetoideae of clade III (red) (Figure 2a), whereas lupulin A has potential antibacterial activity [42] being commonly found in species of clade IV subfamilies, Ajugoideae and Scutellarioideae [42,43,44,45] (Figure 2 and Figure 3).

By examining the NRS descriptor (Figure 2a), it was found that diterpene **5** (Figure 3), which is known as scutalpin L [46,47], presented the highest value for this descriptor, having in its molecule four ring systems, occurring in the subfamily Scutellarioideae of clade IV. Diterpene **6** (crassifol) [48] of the subfamily Nepetoideae shows a null value for the NRS descriptor because it has an acyclic structure (Figure 3).

This confirms that there is a chemical profile of diterpenes, which shows that the subfamilies of clade IV present diterpenes with more ring systems and that the subfamily Nepetoideae (clade III) has molecules rich in hydroxyl groups attached to aromatic and nonaromatic groups.

**Figure 4:**
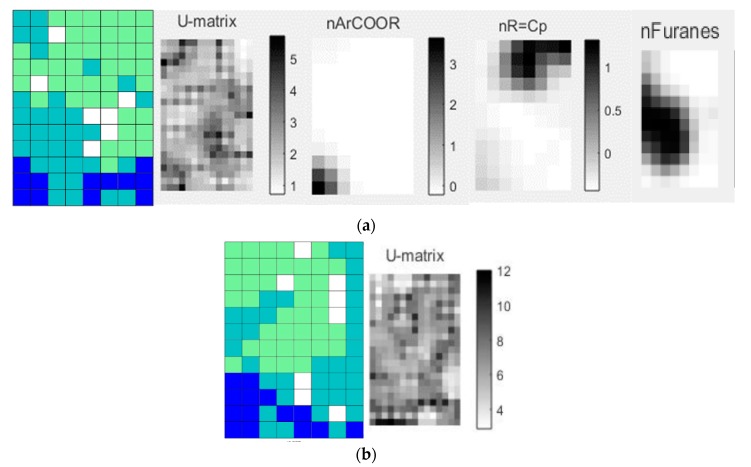
Self-organizing map obtained by the classification of the subfamilies Aju (light blue), Lam (green), Scu (dark blue) and generated descriptors: (**a**) SOM → molecular descriptors; U-matrix; nArCOOR; nR = Cp and nFuranes. (**b**) SOM → fingerprint and U-matrix.

The SOM generated to analyze the correspondences of the 2381 diterpene botanical occurrences of the clade IV subfamilies (Aju, Lam and Scu) resulted in a total hit rate of 91.4% (Table 5). It is also observed that the subfamily Lam presents the best hit rate with 94.8% and the largest number of occurrences and compounds of clade IV; its structural diversity in terms of diterpenes is shown in the SOM (Figure 4). The subfamily Scu shows a hit rate of 81.3%, revealing a clear separation of these subfamilies because all the subfamilies present an accuracy greater than 80%.

Using fingerprint, rates of accuracy were observed close to those obtained using the molecular descriptors; the subfamily Lam had the same hit rate 94.8% in the fingerprint (Table 5). This information supports a good SOM rating performance even when using two different types of descriptors.

Table 6 shows a significant correspondence in the training and test sets of the Aju, Lam and Scu subfamilies. Once more, the AD was reliable for more than 99% of the predictions of the test set. Lamioideae have higher match values: 95.9 and 94.1% for the training and testing, respectively. Scutellarioideae shows lower matching values in the training models with a mean of 76.2% and similar performance in the test results (68.1%). All the total training and test results show a level of significance higher than 60%.

Chemotaxonomy analysis was also performed using other machine learning algorithms, i.e., support vector machine (SVM), which is a supervised machine learning algorithm, and k- nearest neighbors (k-NN), which is an instance-based algorithm. The results are shown in Table 7 for the analysis performed on the SOM by subfamilies belonging to clade IV. It can be observed that, as in the SOM, the models generated with SVM and k-NN obtained very similar results, with high hit rates. The applicability domain (AD) was reliable for over 99% of the test set predictions for all algorithms used: SOM with molecular descriptor, SOM with fingerprint, SVM and k-NN.

In analyzing the SOM and descriptors obtained only from clade IV, the diterpenes of the Ajugoideae, Lamioideae and Scutellarioideae subfamilies that make up this clade were used (Figure 4a). In the map, we can see that there is a proximity between Lam (green) and Aju (light blue), as well as Aju (light blue) with Scu (dark blue), therefore, the pattern of the botanical occurrence of diterpenes does not corroborate with the phylogenetic classification proposed by Li et al. [3], who report that Lam (green) would be closer to Scu (dark blue) than Aju (light blue).

As shown in Figure 4, the self-organizing map obtained by fingerprint showed similarity in the separation of diterpenes when compared to the map obtained by the fragment descriptors. 

Analyzing the descriptors shown in Figure 4a, in the black color for higher values, one realizes that the diterpenes of the Scu subfamily display a high value for the nArCOOR (number of aromatic esters) descriptor; secondary metabolites of subfamily Lam show high values in the descriptor nR = Cp (number of primary C terminals—sp^2^) and the subfamily Aju has molecular structures with higher values of the descriptor nFuranes (number of furans).

**Figure 5:**
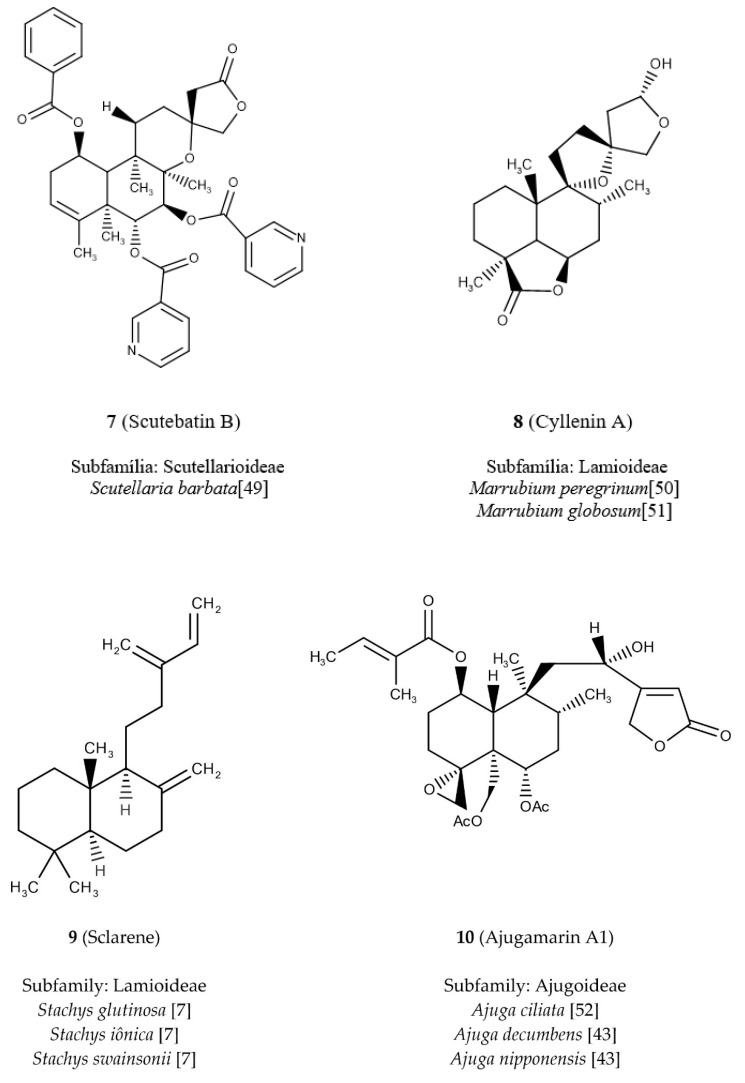

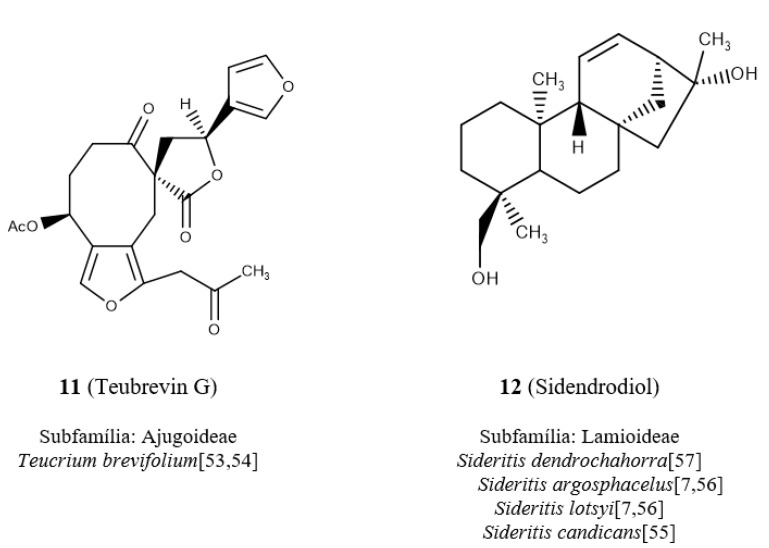
Chemical structures of diterpenes located in SOM and their respective botanical occurrences.

The diterpene **7** (Figure 5) shows the highest value for the nArCOOR descriptor because in its structure it has three aromatic esters. It is commonly known as scutebatin B [49], being found in the subfamily Scutellarioideae (dark blue) (Figure 4a), and the study of its isolation verified its inhibitory effects on the production of nitric oxide aromatic esters induced by lipopolysaccharide in macrophages [49]. We can observe in the descriptor nArCOOR that the white spaces are formed by regions of smaller values, being related to the diterpenes of Lamioideae (green) and Ajugoideae (light blue) (Figure 4a). Thus, we have as example diterpene **8** (Figure 5), known as cyllenin A [50,51], which does not have aromatic ester groups and belongs to the subfamily Lamioideae.

We investigated the highest value reported in the descriptor nR = Cp, which was attributed to diterpene **9** (Figure 5) which is known as sclarene [7]; with three sp^2^ terminal carbons, this diterpene occurs in the subfamily Lamioideae (green) (Figure 4a). The lowest value of the descriptor nR = Cp corresponds to the diterpene **10** (Figure 5), which does not present any terminal carbon sp^2^ and is located in the subfamily Ajugoideae (light blue). Diterpene **10** is known as ajugamarin A1 [43] and shows a potential neuroprotective effect [52].

The diterpene **11** (Figure 5), teubrevin G [53,54], presents the highest value for the nFurane descriptor because there are two furan rings. Observing the descriptor in the black region, which represents higher values, and comparing with the map matches with the same region in which the diterpenes of Ajugoideae occupy confirms that this diterpene occurs in the subfamily Ajugoideae. The diterpene **12** (Figure 5), known as sidendrodiol [7,55,56,57], belongs to the species that occur in the subfamily Lamioideae and does not have furan groups.

The Lamiaceae family includes the genus *Scutellaria*, which belongs to the subfamily Scutellarioideae, and has a cosmopolitan distribution of around 360 species worldwide and in different climatic regions. A majority of its growing species in Asia have a long tradition in Chinese folk medicine [46]. Several studies indicate that diterpenes are commonly found in these species. *Isodon,* belonging to the Nepetoideae subfamily, is another genus with the same cosmopolitan distribution and concentrating the largest distribution in Asia. Several descriptions of species of this genus are reported, however, they have quite different chemical substances from those found in the Scutellarioideae subfamily as we can verify the execution rate of the records of SOMs analyzed in clade III and clade IV [58].

## 3. Materials and Methods

### 3.1. Diterpenes Database

A database of diterpene molecules isolated from the Lamiaceae family was constructed based on a literature review that was performed using an electronic search in SciFinder (https://scifinder.cas.org/) and Web of Science (https://clarivate.com/products/web-of-science/), covering articles published between the years 1980 and 2017. Subsequently, the database was made available in the web tool SistematX [32]. The chemical structures, SMILES codes, names of the compounds (chemical and common), bibliographic references, as well as specific information for taxonomic classification (from family to species) and the geographical location of the species from which the compounds were isolated were compiled, and the total number was calculated instantaneously.

### 3.2. Molecular Descriptors

For all diterpene structures, SMILES codes were used as input data for Marvin and ChemAxon (http://www.chemaxon.com/). Then, Standardizer software (http://www.chemaxon.com/) was used to convert the various chemical structures into custom canonical representations, add hydrogens, aromatize, generate 2-D structures and save the compounds in SDF format. After processing in the Standardizer software, the 2-D structures of the compounds were used as input data in the Dragon 7.0 program [24]. This program has the capacity to calculate 5270 molecular descriptors covering several theoretical approaches and distributing the descriptors into 30 logical blocks. In Dragon 7.0, the coordinates of the atoms of each molecule were selected and then 301 molecular descriptors distributed in three blocks were calculated: ring descriptors, functional groups and atom centralizers [24]. Ring descriptors are numerical quantities that encode information about the presence of rings in a molecule; functional groups are groups of atoms with characteristic and specific reactivity; centered descriptors are defined as the number of specific types of atoms in a molecule [59].

The constant variables were excluded for each block of descriptors and those that presented a different value in the series. The remaining 119 molecular descriptors that were submitted to statistical analysis were 32 rings, 39 functional groups and 37 atom-centered fragments.

In Dragon 7.0, the coordinates of the atoms of each molecule were selected and then 1024 fingerprints descriptors were calculated with the following atom options: atom type, aromaticity, attached hydrogens, connectivity (total), total bond order, connectivity (no H), ring memberships in smallest set of smallest rings (SSSR), smallest ring size in SSSR and bond order.

### 3.3. SVM and kNN Models

The Knime 3.6.2 software (Knime 3.4.0 the Konstanz Information Miner Copyright, 2003–2017, www.knime.org) was used to perform all of the following analyses. The descriptors and class variables were imported from the software Dragon 7.0, and for each one the data were divided using the “partitioning” node with the “stratified sample” option to create a training set and a test set, encompassing 80% and 20% of the compounds, respectively. Although the compounds were selected randomly, the same proportion of active and inactive samples was maintained in both sets. Two models were generated using the support vector machine algorithm (SVM) and the k-nearest neighbors algorithm (k-NN). The models were modeled following a 5-fold external cross-validation. 

SVM is a supervised machine learning algorithm that analyzes data and recognizes patterns [60,61].

The parameters selected for the SVM for all generated models were polynomial, power 1.0, bias 1.0 and gamma 1.0.

k-NN consists of instance-based machine learning, i.e., the function is approximated only locally (neighbors) and the entire calculation is postponed until classification [62,63]. It is a technique that gives weight to neighbors’ contributions, so that the nearest neighbors contribute more to the average than do the more distant ones [62,63]. The parameter selected for the SVM for all generated models was k = 3.

### 3.4. Self-Organizing Maps

The previously selected molecular descriptors were analyzed with SOM Toolbox 2.0 [37,64]. The SOM Toolbox is a set of MATLAB functions that can be used for the elaboration and implementation of neural networks because it contains functions for the creation, visualization and analysis of SOMs [37,64]. The data set was presented to the network before any adjustment was made. Subsequently, the data group was partitioned according to the regions of the map weight vectors at each training stage. Then, the correct prediction of these sets and the correct total prediction of the compounds were evaluated. In the most relevant models, the set was divided into training and testing to assess predictability. The training and test performances were evaluated by calculating the proportion of the number of samples classified correctly by the SOM. For each map, 5-fold cross-validation was performed, with data being partitioned into 80% training and 20% test (Table 8 and Table 9). In the SOM, the sites containing molecules for each descriptor were identified to show existing chemical patterns. For the AD, which is defined as a theoretical region of the physicochemical and response space of the model that allows one to estimate the uncertainty in the prediction of a particular compound based on how similar it is to the training compounds employed in the model [60], the AD Enalos node in the Knime 3.7.1 software was used [61]. The AD based on the Euclidean distances was used to identify compounds in the test set for which predictions may be unreliable if the values are higher than AD = d + Zσ, where d and σ are average Euclidian distance and standard deviation, respectively, of the set of samples in the training set that have lower Euclidian distance than the average values of all samples in the training set. The parameter Z is an empirical cut off value, 0.5 was used as the default.

## 4. Conclusions

The database of the present work presents a great diversity of diterpenes of the family Lamiaceae that were available in the web tool SistematX (https://sistematx.ufpb.br), with more than 4115 molecules distributed in 639 species of 66 genera and eight subfamilies, totaling more than 6386 botanical occurrences. The SOMs obtained from the Lamiaceae subfamilies, using molecular descriptors, separated the subfamilies with high accuracy rates (>80%) and corroborate previous phylogenetic studies by Li et al. [3]. Thus, SOMs based on physicochemical properties encoded from diterpenes are a useful tool to search for structures with defined characteristics and can be used, for example, in the search for diterpenes with potential biological activity using taxonomic and geographic data.

## Figures and Tables

**Table 1:** Lamiaceae subfamilies listed according to Li et al. [3]. Abbreviations, botanical data, number of diterpenes and chemical occurrences added and used in SistematX (https://sistematx.ufpb.br).

Subfamily	Acronym	Genera	Species	Diterpenes	Occurrences
Ajugoideae	Aju	7	99	580	856
Callicarpoideae	Cal	1	14	71	86
Lamioideae	Lam	23	188	601	1183
Nepetoideae	Nep	30	289	2433	3644
Peronematoideae	Per	1	1	7	7
Premnoideae	Pre	2	7	85	92
Scutellarioideae	Scu	1	31	286	342
Viticoideae	Vit	1	10	130	169
Total		66	639	4115	6379

**Table 2:** Results of the self-organizing map with the values of the occurrences and the number of correct hits for clade III (Nep) and clade IV (Aju, Lam and Scu) of the family Lamiaceae, using the descriptors generated by the program DRAGON 7.0 [24].

			Molecular Descriptors		Fingerprint	
Subfamily	Diterpenes	Occurrences	No. of Hits	% of Hits	No. of Hits	% of Hits
Clade III	2433	3644	3252	89.2	3366	92.3
Clade IV	1453	2381	1948	81.8	2031	85.3
Total	3886	6025	5200	86.3	5397	89.5

**Table 3:** Summary of results of training and test match (%) of clade III (Nep) and clade IV (Aju + Lam + Scu).

**Subfamily**	**Train Set 1**	**Train Set 2**	**Train Set 3**	**Train Set 4**	**Train Set 5**	**Average**
Clade III	91.4	87.1	89.5	88.5	89.4	88.6
Clade IV	79.9	84.8	80.1	83.2	82.3	82.1
Total	86.9	86.2	85.8	86.4	86.6	86.4
**Subfamily**	**Test Set 1**	**Test Set 2**	**Test Set 3**	**Test Set 4**	**Test Set 5**	**Average**
Clade III	89.4	87.7	89.8	87.5	87.2	88.3
Clade IV	76.9	84.2	84.2	78.6	81.1	81.0
Total	84.5	86.3	87.6	84.0	84.8	85.4

**Table 4:** Summary of test match (%) corresponding to the results obtained from 5-fold models using self-organizing map (SOM), support vector machine (SVM) and k-nearest neighbors (k-NN) algorithms for clade III (Nep) and clade IV (Aju + Lam + Scu).

Subfamily	SOM Average	SOM fingerprint Average	SVM Average	k-NN Average
Clade III	88.3	88.1	92.2	96.2
Clade IV	81.0	80.0	88.9	92.2
Total	85.4	89.5	90.9	94.6

**Table 5:** Results of the self-organizing maps with the occurrence values and the number of correct hits for the subfamilies belonging to clade IV (Aju, Lam and Scu), using the descriptors generated by the Dragon 7.0 program.

			Molecular Descriptors		Fingerprint	
Subfamily	Diterpenes	Occurrences	No. of Hits	% of Hits	No. of Hits	% of Hits
Aju	580	856	776	90.7	742	86.6
Lam	601	1183	1122	94.8	1122	94.8
Scu	286	342	278	81.3	320	93.5
Total	1467	2381	2176	91.4	2184	91.7

**Table 6:** Summary of the results of training and test match (%) of Aju, Lam and Scu.

**Subfamily**	**Train Set 1**	**Train Set 2**	**Train Set 3**	**Train Set 4**	**Train Set 5**	**Average**
Aju	86.6	90.2	92.8	88.6	87.6	89.2
Lam	96.4	96.2	95.7	94.6	96.5	95.9
Scu	78.4	75.9	67.2	76.6	82.8	76.2
Total	91.4	91.1	90.6	89.9	91.3	90.9
**Subfamily**	**Test Set 1**	**Test Set 2**	**Test Set 3**	**Test Set 4**	**Test Set 5**	**Average**
Aju	93.0	90.1	89.5	90.6	82.5	89.1
Lam	93.6	91.1	96.6	95.8	93.2	94.1
Scu	63.8	67.6	54.4	70.6	84.1	68.1
Total	89.1	87.4	88.0	90.3	88.0	88.6

**Table 7:** Summary of test match (%) corresponding of the results obtained 5-fold models using SOM, SVM and k-NN algorithm of the subfamily Aju, Lam and Scu.

Subfamily	SOM Average	SOM Fingerprint Average	SVM Average	k-NN Average
Aju	89.1	83.6	94.6	95.3
Lam	94.1	87.3	97.0	97.6
Scu	68.1	95.8	87.4	88.0
Total	88.6	92.7	94.7	95.4

**Table 8:** Summary of the five different training and test sets related to SOM obtained with diterpenes from clades III (Nep) and IV (Aju, Lam and Scu).

	Train Set		Test Set		Total
	Train	% Total	Test	% Total	
Clade III	2915	80	728	20	3643
Clade IV	1905	79.9	477	20.1	2382

**Table 9:** Summary of the five different sets of training and test related to SOM obtained with diterpenes only from Clade IV (Aju, Lam and Scu).

	Train Set		Test Set		Total
	Train	% Total	Test	% Total	
Aju	684	79.9	172	20.1	856
Lam	947	80	236	20	1183
Scu	273	79.8	69	20.2	342

## Abbreviations

SOM	Self-Organizing Map
ANNs	Artificial Neural Networks
NuBBE	Bioassay Nucleus, Biosynthesis and Ecophysiology of Natural Products
DNP	Dictionary of Natural Products
Marinlit	Natural marine products
Aju	Ajugoideae
Cal	Callicarpoideae
Lam	Lamioideae
Nep	Nepetoideae
Per	Peronematoideae
Pre	Premnoideae
Scu	Scutellarioideae
Vit	Viticoideae
NRS	Number of ring systems
O-056	(Alcohol) atom-centered fragments
nArOH	Number of aromatic hydroxyls
nArCCOR	Number of aromatic esters
nR = Cp	number of primary C terminals - sp2
nFuranes	number of furans
